# Supplementary material for: Are carbon emissions trading and green financial instruments synergistic? -Comprehensive quantitative research based on content analysis
Source: PLoS One. 2024 Mar 7;19(3):e0298601. doi: 10.1371/journal.pone.0298601 (PMC10919601; doi:10.1371/journal.pone.0298601)
Supplement: S1 Data — (ZIP) [file pone.0298601.s001.zip › Data/地级市Z_F工作报告.zip!/╣π╢1⁄2/╔╟═╖╩╨/└┤╫╘╣1⁄2╓┌║┼í╛┬φ┐╦╩2╛▌═°í┐_2005.docx]

各位代表：

我代表市人民政府向大会作政府工作报告，请予审议，并请各位政协委员和其他列席人员提出意见。

2004年工作回顾

2004年，是我市经济社会在新的起跑线上平稳健康、加快发展的一年。一年来，市政府在省委、省政府和中共汕头市委的正确领导下，在市人大及其常委会和市政协的监督支持下，树立和落实科学发展观，认真贯彻市委工作会议精神，求真务实，奋力开拓，不断化解经济社会发展面临的困难和矛盾，全市经济实现较快恢复性增长，社会稳定和谐，顺利完成了市十一届人大二次会议确定的主要任务。

———国民经济稳步发展

全年完成生产总值603．8亿元，比上年增长11．1％，增长速度近6年来首次达到两位数。按可比口径计算，一般预算收入24．65亿元，增长18．65％。

工业主导地位日益显现。实现工业总产值1076亿元，增长13．1％，首次突破千亿元大关。工业对生产总值增长的贡献率达到58．4％。规模以上工业增长15．1％，占全部工业总产值的50．9％。金威啤酒、粤东磁电等60多个重点工业项目建成或投产，工业用电量大幅攀升。工业经济结构继续优化，八个支柱产业的产值占全市工业总产值的比例比上年提高8．5个百分点。高新技术产品产值160亿元，比增14．3％，电子信息和机械装备产业成为工业增长的亮点。传统产业改造升级力度加大，新增工业技改项目75项。园区经济和产业集群发展加快。名牌带动战略取得新进展，新增中国名牌产品4个、省著名商标18件、省名牌产品15个，潮南区峡山、两英、陈店镇分别被授予“中国家居服装名镇”、“中国针织名镇”、“中国内衣名镇”称号。新增国家免检产品1个，标准化工作被国家标准化委评为先进。建筑业完成施工产值106．8亿元，增长12．6％。

农业基础地位得到加强。农业总产值80．6亿元，增长1．1％。粮食播种面积110．2万亩，总产量53．4万吨，完成省政府下达的粮食工作考评指标。丢荒土地的复耕复种工作力度加大。编制优势农业区域化发展规划和农业科技发展规划纲要，农业结构继续调整优化。农业产业化经营步伐加快，新增国家级农业龙头企业2家。新制订省市农业地方标准34项，设立省市农业标准化示范区9个，新获得国家级无公害农产品认证90个、“绿色食品”认证10个，金平浦片区成为省级农业科技园。农田水利基础设施建设得到加强，城乡防灾减灾水利建设工作被省评为年度先进。牛田洋海堤达标加固、秋风岭水库除险加固等工程顺利推进，共维修加固江河堤围137公里，除险加固山塘水库41宗。实施人工增雨和水源调配等措施，有效缓解持续干旱的不利影响。动物防疫工作力度加大，抗击高致病性禽流感取得阶段性胜利。海水养殖产量增长10％，新增省级无公害养殖基地4个。新增造林面积2．36万亩，海岸湿地自然保护区成为国内首个国际湿地示范区。

第三产业加快发展。消费市场日趋繁荣，社会消费品零售总额292亿元，增长14．8％。消费结构出现新变化，住房、汽车、通讯产品和旅游休闲等消费热点增长较快。会展业持续兴旺，汕头国际毛衫内衣交易会、塑料博览会和澄海国际玩具工艺博览会等大型展会相继举行。旅游业稳步发展，接待过夜旅游者283万人次。南澳岛成为全国第一个AAAA级海岛旅游区。汕头、潮阳本地电话网顺利并网。金融、证券、保险业稳步发展。金融支持经济力度加大。年末金融机构本外币存款余额887亿元，比上年末增长11％。交通运输业进一步发展，货运量1605万吨，增长8．3％；港口货物吞吐量1578万吨，增长7．4％，其中集装箱28．5万标箱，增长27．9％。

———对外开放步伐不断加快

招商引资取得新进展。新引进外商直接投资项目78个，外商投资企业增资扩产75家。实际吸收外商直接投资金额7828万美元，增长50．6％。成功举办“10·19”招商引资经贸活动，共有208个项目分别签约、开工和投产，总投资额136亿元。

外贸出口保持快速增长。外贸进出口总额41．8亿美元，增长24．9％。出口25．5亿美元，增长34．5％。进口16．3亿美元，增长12．4％。服装、玩具、塑料制品和机电类产品出口大幅增长；努力消除反倾销影响，水产品出口与上年持平。民营企业出口8．3亿美元，增长53％，成为外贸出口的生力军。

对外交流合作进一步扩大。成功举办汕港经贸合作交流会和海峡两岸高新技术产业交流协作活动、汕头（澳门）美食文化节。汕头—香港国际邮轮航线开通。赴欧洲和东南亚开展经贸活动，成功举办汕头（巴黎）投资环境推介恳谈会。赴广西、湖南参加泛珠三角经贸协作活动，赴东北三省开展经贸合作，汕头对外影响力日益扩大。

———基础设施和市政建设有新突破

一批事关长远发展的规划编制顺利推进。城市发展概念规划通过专家评审，进入最后综合成果编制阶段。“三条经济带”的规划论证逐步展开，工业经济带规划完成市级评审，城市经济带完成堤路选线。潮南区分区规划编制完成。城市管道燃气、排水系统、绿地系统、夜景照明等一批专项规划相继完成。广澳港区详细规划修编通过专家初审。

一批重点工程项目顺利推进。全社会固定资产投资额132亿元，增长10．7％。外砂大桥、莲阳大桥扩建和嵩山路北段、韩江路等一批项目开工建设。珠池港区二期工程、石大桥北引道、国道324线峡山至陈店路段、省道237线和平至谷饶路段、24个行政村43公里水泥路等项目基本建成。长平路与华山路交叉路口“水浸街”改造等工程竣工。新建续建110千伏及以上输变电项目34个。金凤路桥、金鸿公路、广澳港区一期工程、汕头港主航道及外拦门沙二期工程等重点项目继续推进。

———改革创新迈出新步伐

各项改革继续深化。出台国有企业产权改革操作规程等一批文件，国企改革步入规范化轨道。全市公有制企业实施产权改革77家；其中，市直公有制企业完成改革27家，安置职工2720人。国有粮食企业改革全面启动。农村税费改革稳步推进，减负率达92．7％。并村工作顺利完成。物价改革继续推进。路桥收费年票制改革经省政府批准顺利实施。

民营经济实力不断壮大。民营工业实现产值866亿元，增长19．4％，占全市工业总产值的80．5％。民营企业做大做强，2家企业进入全省百强民营企业行列，宜华木业成为汕头第一家上市的民营企业。8家企业获“中国优秀民营科技企业奖”，50家企业成为省级民营科技企业。

市场体系进一步健全。土地、技术、劳动力、人才等要素市场不断完善。积极开展食品药品、市场物价等专项整治，加大力度打击制假贩假、走私贩私等经济违法犯罪活动，社会信用体系逐步完善，市场经济秩序不断规范。

积极解决经济领域的难点问题。市商业银行最终处置的准备工作加紧进行，制订多套工作预案，资产保全清收工作力度加大。争取上级大力支持，促使解封潮阳港区和关埠港区资产。加强用电管理，降低商业电价。统筹调剂用地需求，加大闲置土地的清理力度，清理各类开发区63个，初步整合为7个较大规模的工业区。

———精神文明和民主法制建设进一步加强

思想道德建设和精神文明建设取得新成效。“三个代表”重要思想学习活动深入开展。群众思想道德素质进一步提高。未成年人思想道德建设得到加强。军民共建精神文明活动继续推进，双拥模范城创建成果进一步巩固。开展全民读书月活动，群众性精神文明创建活动取得新进展。

民主建设继续推进。自觉坚持向市人大及其常委会报告工作和向市政协通报情况。人民代表议案（建议）74件、政协委员提案181件全部办理完毕，办理结果满意和基本满意率有所提高。政务、村务、厂务公开不断推进。

法制建设步伐加快。深化行政管理体制改革，政府社会管理和公共服务职能得到强化。完成第三轮行政审批项目清理，削减审批事项161项。制订实施依法行政五年规划。推进立法科学化、民主化，公开征集立法项目，试行立法听证会制度。提请市人大常委会审议的法规草案4件，颁布市政府规章5件、规范性文件42件。推行行政执法责任制和评议考核制，行政执法、执法监督和行政复议工作得到加强。全民普法教育不断深化，依法办事的氛围日渐浓厚。

廉政建设和作风建设得到加强。纠风工作深入开展，机关作风不断改进。各级政府公开作出并认真履行廉政承诺，廉政责任制得到落实，违法违纪人员受到查处，政府廉洁从政的形象进一步改善。

社会保持和谐稳定。加大矛盾纠纷排查调处工作力度，高度重视人民群众来信来访工作，信访办理质量有所提高。加强对群体性事件的防范处置，确保了社会政治稳定。打击暴力性犯罪、“两抢两盗”犯罪专项斗争持续开展，社会治安综合治理继续推进，社会治安秩序稳定。安全生产工作得到加强。

———社会各项事业取得新进展

科技创新成果显著。新增科技成果76项，获省科学技术进步奖8项，推广转化科技成果109项。以企业为主体的技术创新体系逐步完善，新组建3个省级工程技术研发中心，新增国家级、省级高新技术企业32家。信息化工作取得新进展。新增专利授权1650件，获全国专利优秀奖2项，涌现一批全省知识产权试点区域、园区、街道以及省技术创新试点示范镇，金平区成为全国科技工作试点县（区）。

教育事业蓬勃发展。以县为主的基础教育管理体制进一步落实，“普九”成果巩固提高。中小学布局调整工作逐步推进，新增学位5万多个，“入学难”问题得到有效缓解。潮阳区镇二小学等“大班额”问题得到解决。高中入学率有所提高，优质学位增加。教育教学质量进一步提高，高考成绩继续名列全省前茅。职业技术教育和成人教育有新突破，民办教育健康发展。汕头职业技术学院办学规模不断扩大，汕头大学办学水平进一步提高。

文化事业和文化产业持续发展。文化大市建设步伐加快，全市文化活动蓬勃开展。成功承办第十届全国美展水彩、粉画展。基层文化设施建设落后状况有所改善，龙湖区获得“南粤锦绣工程”文化先进区称号。市博物馆新馆启用，林百欣图书馆装修工程顺利推进。文化产业初具规模，文化市场在规范中发展。文化艺术、新闻出版、广播电视、社会科学等在经济社会发展中发挥积极作用。

卫生、体育等事业稳步发展。市疾控中心工程奠基，传染病疫情和突发公共卫生事件监测网络进一步完善。参加农村合作医疗101．8万人，覆盖率32％。国家卫生城市创建成果继续巩固，顺利通过省复检。汕头籍运动员获得2枚奥运会金牌，实现奥运金牌零的突破。成功承办第20届全球华人篮球邀请赛等大型赛事，国际渡海节等群众性体育活动广泛开展。国防动员和民兵预备役工作得到重视和加强。社区建设不断推进，完成“星光计划”第三批项目。老龄事业加快发展。荣获“全国残疾人工作示范城市”称号。区、镇（街道）勘界工作基本完成。档案、保密、方志、人防、气象、地震、妇女儿童等各项事业有新进步。统计、审计、物价、外事、侨务、对台、宗教等工作取得新成绩。

人口计生工作初步扭转被动局面。推行农村部分计划生育家庭奖励制度，纯二女户结扎、依法征收社会抚养费、查处党员干部违法违规生育等三项重点工作扎实推进。建立流动人口计划生育管理与服务新机制，基层计生基础建设逐步加强。完成省下达的人口控制指标，人口自然增长率6．23‰。

环境和资源保护工作得到加强。积极创建国家园林城市，植树44．8万株。重点流域、区域环境整治继续推进，龙湖沟引韩冲污一期工程基本完成，环保专项行动成效明显。生态示范建设取得新进展，新创建8个生态示范区，南澳成为国家生态示范区。殡改工作进一步加强。

人民生活有所提高。中心城区居民人均可支配收入9930元，增长9．1％；农村居民人均纯收入4089元，比上年略有增加。城乡居民本外币储蓄存款余额681亿元，增长12％。“十项民心工程”扎实开展，认真解决关系民生的热点难点问题。全面落实再就业目标责任制，兑现再就业优惠政策，城镇新增就业6．6万人，城镇登记失业率为3％，国有企业下岗失业人员再就业率达到65％以上。推进和完善医疗、生育保险，社会保险覆盖面不断扩大。实行社会保障市级统筹，企业退休人员社会化管理稳步推进。城乡最低生活保障和社会救助制度得到落实，法律援助走在全省前列。25个集体经济年纯收入3万元以下的行政村实现脱贫。潮阳、潮南引韩工程动工建设，加快解决农村群众饮水难问题。

各位代表！过去的一年，我们突出“七个坚持”：坚持以科学发展观为指导，抓住宏观调控的机遇，加快经济结构调整和经济增长方式转变；坚持求真务实，在奋进中加快发展，在加快发展中解决问题，在解决问题中凝聚人心；坚持园区带动、品牌兴业、产业集群，走新型工业化发展路子；坚持扩大出口、扩大投资、扩大消费，拉动经济增长；坚持规划论证、招商引资、落实项目，为今后加快发展创造条件；坚持创新体制、完善机制、加强管理、理顺关系，优化发展环境；坚持集中力量解决热点难点问题、办好民心工程，维护好、实现好人民群众的根本利益。通过以上有效措施，确保了全年经济和社会发展预期目标的完成。上述成绩的取得，是省委、省政府和市委正确领导的结果，是各级人大及其常委会和人民政协监督支持的结果，是全市人民同心同德、奋力拼搏的结果，也是社会各方面支持和帮助的结果。在此，我代表汕头市人民政府，向全市广大工人、农民、知识分子、干部职工、驻汕人民解放军、武警官兵、人民警察和各界人士表示崇高的敬意！向为全市现代化建设作出贡献的广大劳动者和建设者表示衷心的感谢！向关心和支持汕头建设的港澳台同胞、海外侨胞和国际友人表示衷心的感谢！

我们清醒地看到，我市经济社会发展还存在着不少困难和问题。主要是：规模以上工业比重偏低，经济结构不够合理；投资增速偏低，经济持续增长动力不足；区域发展不平衡，“三农”问题仍很突出；市商业银行的最终处置任务十分艰巨，社会管理、环境保护任务很重；财政收支矛盾十分突出；人口和就业压力很大，城乡居民收入增长不快；投资软环境仍不如人意，行政效能有待提高等等。对这些问题，我们将认真采取措施加以解决。

2005年工作安排

2005年，是全面落实科学发展观，调整结构，抢抓机遇，攻坚克难，加快发展的关键一年。新的一年，经济全球化步伐加快，国际投资和贸易继续扩大，世界经济持续增长；我国经济正处于新一轮增长周期，经济结构不断调整优化，转变经济增长方式的步伐加快，国民经济将持续稳定健康发展，这为我们加快发展提供了有利的外部环境。同时，国际环境的不确定因素仍然存在，国家继续加强和改善宏观调控，这对我市加快发展提出了更高的要求。形势逼人，机遇催人，新的一年是充满希望和挑战的一年。我们一定要以强烈的责任意识、机遇意识和饱满的热情、务实的作风，推动汕头经济社会走上发展的快车道。我们一定要立足全局和着眼长远，以战略的眼光和统筹协调的思维，积极谋划汕头更加美好的未来！

2005年政府工作的总体要求是：以邓小平理论和“三个代表”重要思想为指导，全面树立和落实科学发展观，认真贯彻中央经济工作会议和省委九届六次全会、市委八届四次全会精神，坚持以发展为第一要务，围绕建设经济强市和区域性中心城市的奋斗目标，抢抓机遇，乘势而上，深化改革，扩大开放，加快经济结构调整，加快经济增长方式转变，提高经济增长质量，不断增强综合经济实力、地区竞争力和发展后劲，不断提高人民生活水平，不断推进物质文明、政治文明和精神文明建设，努力建设和谐社会，促进经济快速健康发展和社会全面进步。

按照上述总体要求，做好全年工作，必须努力做到“六个统一”：一是把谋求经济增长与调整优化产业结构统一起来，在加快经济发展的同时促进产业协调发展；二是把扩大经济总量与提高经济质量统一起来，在做大经济堆头的同时促进经济增长方式的转变；三是把立足当前和着眼长远统一起来，在抓好当前工作的同时以战略眼光谋长远、谋未来；四是把发展内源型经济和外源型经济统一起来，创新体制机制、深化改革、扩大开放；五是把加快经济发展与可持续发展统一起来，推动经济与环境、人口、资源全面协调发展；六是把经济与社会协调发展统一起来，努力构建和谐汕头。

按照上述总体要求，做好全年工作，必须着力寻求“四个突破”：一是狠抓招商引资和园区带动，力争在招商引资和重大项目引进上有新突破；二是狠抓规划建设和投资拉动，力争在基础设施建设和固定资产投资上有新突破；三是狠抓扩大出口和扩大消费，力争在增强发展活力上有新突破；四是狠抓经济社会管理和行政效能建设，力争在优化发展环境上有新突破。

2005年全市经济社会发展的主要预期目标是：生产总值增长11％，一般预算收入增长11％，社会消费品零售总额增长13％，固定资产投资增长12％，外贸出口增长11％，居民消费价格总水平上涨控制在3．8％左右，城镇登记失业率控制在3．5％以内，人口自然增长率控制在7‰以下，城镇居民人均可支配收入实际增长8％，农村居民人均纯收入实际增长4％。

为实现上述目标，必须抓好以下工作：

一、加快工业结构调整步伐，走新型工业化道路

推动产业升级。尽快出台《关于加快工业发展的决定》，加强产业规划和产业引导，不断优化产业布局。大力发展新型产业，使工业产业结构逐步由加工型、轻型向高级化、适度重型化转变。继续发展传统产业，做大做强支柱产业，推动支柱产业的集群和主导产品的配套，不断增强产业竞争力。加快发展装备制造业，重点发展食品、包装、印刷、塑料机械制造、输配电及控制设备制造和船舶制造业。

壮大规模企业。下大力气扶持和规范中小企业，引导其向规模以上企业发展，提高规模以上企业的比重。从土地供应、增资扩产、技术改造、品牌培育、出口通关、政府服务等方面对龙头企业给予倾斜，增强龙头企业的辐射和带动作用。重点做好投资额1000万元以上的工业投资项目的跟踪服务工作，扩大工业投资规模。

发展园区经济。通过特色的产业定位、良好的产业生态、完善的产业链条、配套的基础设施、适度的规模效应，提高园区承载能力，推动产业集群发展。重点规划建设工业经济带，带动保税区、石化园区和潮阳经济开发试验区、潮南工业区的发展；通过级差地价、路桥收费改革等措施，增强南区各园区的竞争力；加快南区石化园区的规划和报批工作，使南区成为我市发展大工业的载体。继续推进高新区和金平、龙湖民营科技工业园以及澄海岭海工业区规划建设，通过产业和项目选择，做强做优北区工业园区。

推进技术进步和品牌振兴。坚持以信息化带动工业化，应用高新技术和先进适用技术改造提升传统产业。大力扶持拥有自主知识产权和核心技术的企业，积极发展高新技术产业。引导和支持企业做好商标注册、专利申请、质量体系认证、产品认证、原产地标记认证和标准化生产。实施以科技和质量为内在支撑的名牌战略，争取获得更多的国家级、省级品牌，培育发展区域品牌。引导现有的名牌产品做强做大，形成拳头产品。

二、以港兴市，发展壮大临港产业

完善港口规划编制。强化规划战略意识，控制利用好岸线资源，加大中小型泊位、码头区域布局的调整力度。进一步完善广澳港区的详细规划，抓紧编制潮阳、潮南、澄海、南澳港区规划和岸线规划，抓紧做好广澳港区疏港铁路的规划论证。

推进港口码头建设。加快深水航道、深水泊位及其保障系统建设，增强主枢纽港竞争力。重点抓紧广澳港区一期工程、汕头港主航道及外拦门沙整治二期工程，开工建设广澳港区5万吨级航道工程、5万吨级石化泊位。规划广澳港区10至15万吨级深水泊位和30万吨级油码头。尽快盘活潮阳港区资产，引进战略合作伙伴，加快基础设施建设。

培育临港工业。抓住汕头被列为全省规划建设五大石化基地之一的契机，继续抓紧洽谈引进大型石化项目。做好聚苯乙烯、中海油LNG接收站等项目的跟踪服务，加快立项报批。争取濠江涤纶项目尽快开工。加快华能汕头电厂二期工程建设，确保年底发电；全力以赴推进华能海门电厂一期工程前期工作，力争尽快获准立项；大力发展南澳、濠江风力发电等新能源项目。

发展港口服务业和海运业。推进港口体制改革，引入竞争机制，形成国资、外资、民资共同参与港口服务业经营的格局。积极推动区域经济协作，扩大港口经济腹地。积极发展海运业，发展汕头港至盐田港外贸集装箱内支线等国内外航线，大力拓展远近洋运输和水上运输。发挥已有物流平台和保税区的功能作用，大力发展港口物流业。加紧做好燃煤中转基地预选址和论证工作。

三、进一步扩大开放，提高招商引资水平

加强对外经贸合作。发挥侨资民资两大优势，积极利用国际国内两种资源，抓住CEPA实施和泛珠三角区域合作以及中国—东盟自由贸易区建立的契机，主动承接欧美、日韩、港澳台、东南亚的产业转移，承接珠三角和国内先进地区的产业转移，促使外源型经济由小变大。搭建潮商交流合作平台，鼓励海内外潮籍成功人士回乡投资建设。狠抓项目洽谈、引进，强化跟踪服务，做足前期工作，抓紧重大项目立项申报。鼓励企业“走出去”，推动民资、国资与外资的战略合作。组织赴港、台、珠三角专项经贸合作交流活动，继续组织全市性招商引资经贸活动。

创新招商机制。加强与国内外商会的合作，把招商活动由政府主导逐步转变为行业协会、商会主导。整合行政资源和社会资源，通过网上招商、委托招商和园区招商、产业链招商等多种方式，促使招商引资向市场化、产业化、专业化发展。尝试与投资商合作开发园区。建立健全招商引资工作责任制和激励机制，加强企业投资服务工作，健全投资促进机制。

努力扩大进出口贸易。实施科技兴贸、品牌兴贸，提高出口产品的竞争力，转变外贸增长方式，确保出口持续增长。支持引导企业积极应对国际贸易摩擦，引导企业利用国际贸易优惠，保护企业合法权益。扩大民营企业出口，努力开拓中东、俄罗斯、非洲及南美等新兴市场。继续扩大一般贸易出口，促进加工贸易转型升级。充分利用商贸优势和区位优势，做大进口贸易总量。推进“大通关”建设，不断改善通关环境。

四、大力实施投资拉动，加快“三条经济带”规划建设

完善规划编制工作。尽快完成城市发展概念规划综合成果的编制，启动土地利用总体规划修编和新一轮城市规划修编工作。强化规划管理，加强对全市各层次规划的综合协调指导工作，做好专项规划和分区规划、控制性详细规划的编制。

加快“三条经济带”的规划建设。积极开展“三条经济带”规划建设的前期工作，为城市发展、工业发展提供更大的空间和战略腹地。抓紧工业经济带报批工作，争取局部启动建设；加快完成东延城市经济带水文、海洋地质、环境评价等论证工作，抓紧编制堤路规划设计、可研报告并上报立项；积极做好西延生态经济带的前期规划论证。

抓紧基础设施建设。开工建设潮南陈沙公路、省道237线谷饶至贵屿路段改造工程；协力推进汕揭梅高速公路汕头段工程、深汕高速公路东段大修工程进度；重点加快金凤路桥工程、金鸿公路、潮阳揭海公路、国道324线汕头段4座大桥改造等项目的建设；推进农村公路工程建设；抓紧沿海铁路汕头段选线选站和南澳大桥的立项、规划设计工作。加快500千伏汕头变电站1＃主变电工程扩建及一批配网工程建设，逐步解决供电“瓶颈”问题。加快潮阳、潮南引韩供水工程建设。加快南澳引韩工程的前期工作。续建第二过海水管。

推进市政设施建设和旧城改造。完成嵩山北路南段、利安路、韩江路等市政项目的施工建设；改造中山西路、外马路和杏花街。抓紧海滨路中、西段拓宽改造工程前期工作，争取今年动工。加快旧城改造步伐，推进小公园片区改造工程建设，逐步改造“城中村”。争取完成中山公园改造。加大创建国家园林城市工作力度，确保顺利通过国家、省的考核验收。全面加强城市景观规划建设，继续加大城区绿化建设力度，完成主要路段的灯饰工程，美化江、河、海岸景观。

加强城乡防灾减灾工作。逐步建立地震、台风、海潮等自然灾害的监测和防范机制，提高城乡防灾能力。加强重点防灾设施建设，建立包括防汛、防风、防旱、抗震和防火在内的城乡综合防灾体系。完成牛田洋海堤工程，抓紧东里桥闸重建工程、濠江堤围达标建设，抓好榕江金光围和潮阳、南澳城防工程建设，启动潮南南山截流工程。建成新一代多普勒天气雷达系统，提高预防灾害性天气水平。

五、加强“三农”工作，促进农村经济稳步发展

推进农业产业化。加快农业结构调整，大力发展高效农业、特色农业、品牌农业。建设五大优势农业区，重点抓好绿色食品、无公害农产品示范基地和农业标准化示范区建设。做好名优产品原产地保护，健全动植物检验检疫体系和农产品质量检验检测体系建设，加强动植物防疫和病虫害防治工作。在稳定土地承包制的基础上，鼓励农户依法、自愿、有偿流转土地承包经营权，逐步发展适度规模经营。大力扶持发展加工带动型、市场带动型、科技带动型龙头企业，探索建立和发展农民专业合作经济组织。加强农业技术推广体系建设，实施科技兴农。建设一批无公害渔业养殖基地，大力发展生态渔业。争取海门渔港列入国家级中心渔港，抓紧做好云澳渔港列入国家级中心渔港和达濠渔港列入国家一级渔港的前期工作。

提高粮食综合生产能力。全面落实中央关于扶持粮食生产的政策措施，进一步调动农民种粮积极性。鼓励种粮大户发展规模化、产业化生产，扩大优质稻种植面积。加强基本农田保护，落实非农建设用地耕地占补平衡制度。

加强农田基本建设。切实加大资金投入，大兴农田水利基本建设，提高灌溉和防洪排涝能力。加快小型水库加固和机电排灌改造，加强中低产田改造，抓好农业综合开发。

推进农村城镇化。大力加强中心镇建设，带动各建制镇的发展。加快农村工业化，发展城镇服务业，强化城镇化进程中的产业支撑。完善城镇基础设施和公共服务设施，增强产业和劳动力吸纳功能，促进农业人口向城镇转移和农村劳动力向第二、三产业转移。

切实增加农民收入。全面免征农业税，继续清理整顿涉农收费。加强农村土地和集体资产管理，完善农村征地款的使用管理和监督。做好扶贫开发进村入户和对口帮扶贫困村工作。出台扶持措施，加快老区山区和贫困地区脱贫奔康步伐。继续推进信用村建设。

六、加快发展现代服务业，提高商贸业整体水平

大力发展生产服务业。加强专业市场建设，支持澄海区发展玩具物流中心、玩具礼品集市，支持潮阳区、潮南区发展纺织服装、音像制品、化妆品批发市场。加快发展会展经济，办好玩具工艺博览会、塑料博览会等展会，培育汕头会展品牌。大力发展现代物流业和交通运输业，积极发展金融、信息、中介等现代服务业，增强区域辐射功能。主动参与泛珠三角经贸合作和闽粤赣区域协作，鼓励企业参加国内外各种展会，提高汕货的市场占有率。

积极发展生活服务业。大力发展房地产业和餐饮业，培育住房、汽车、教育、休闲、体育等消费热点，提升消费结构。整合旅游资源，加强旅游项目开发和区域合作，发展具有潮汕特色的滨海旅游。积极改造提升传统商贸业，提高第三产业整体水平。优化消费环境，扩大消费需求。抓好市场主要副食品供求状况的日常监测，加强市场价格的监测和预警，保持市场稳定。

七、深化体制改革，增强发展活力

加快国有企业改革步伐。建立完善国有资产管理体制，确保国有资产保值增值。贯彻实施我市国有企业改革工作的意见，规范国企改制和产权交易。加快市直国企的改制步伐，争取全面完成国有粮食企业改革任务。拓宽改革成本的筹措渠道，妥善处置改制企业的债权债务，做好职工安置工作，确保国企改革健康有序推进。

做大做强民营经济。全面落实支持民营经济发展的政策措施，促进内源型经济由弱变强。引导民资进入基础设施、垄断行业、公用事业以及法律没有禁止的行业和领域。支持民营企业体制创新，鼓励民营企业参与国有、集体企业改革。推动民营企业技术创新，扶持发展民营科技企业。加强中小企业服务体系的建设，提供信息、技术、人才和法律上的咨询与援助。

深化投融资体制改革。规范和简化投资审批，落实企业投资自主权。选择一批市政设施和各类特许经营权进行招投标和公开拍卖，运用BOT、TOT形式，实行市场化运作。依法收回一批闲置土地，公开拍卖一批土地使用权，盘活政府资源。积极争取政策性银行贷款、商业银行贷款、国债资金和上级拨款，加大政府性资金投入，多形式吸引社会资本参与基础设施建设。加强组织实施，确保路桥收费年票制改革顺利推进。

八、加强经济管理，提高经济运行质量

强化税收管理。积极培育税源，拓宽税基，优化税收结构。继续清理经济户口，加快税收征管信息化建设，强化重点税源监控，堵塞各类漏洞，严厉打击偷逃骗税，确保应收尽收。增强协税、护税意识，支持税务机关依法治税。

积极推进公共财政体制改革。完善预算编制改革，市直党政机关、事业单位全面实行部门预算。加强非税收入管理，全力清收结欠财政资金。坚持量入为出、收支平衡，从严从紧控制一般性开支。进一步完善国库集中支付制度。扩大政府采购规模和范围，规范政府采购行为。加强财政资金使用的监督、管理和绩效评价。加强审计监督。

加强和改善金融服务。鼓励和引导金融机构加大对地方经济的支持力度，严厉打击各种逃废金融债务的不法行为。积极与国家开发银行合作，探索建立中小企业信用担保体系，提升企业融资能力。引导企业运用资本市场，逐步提高直接融资比重。积极推进农村信用社改革。稳妥推进市商业银行的最终处置工作。

强化经济管理。健全综合治理工作机制，严厉打击制假售假、走私贩私不法行为，维护市场经济秩序。加强对经济运行的监测、预警、分析和协调，提高经济运行质量。科学编制好“十一五”规划，按时保质完成重点专项规划、区域规划的任务。认真组织好经济普查工作。

九、加强资源和环境保护，促进可持续发展

合理开发利用资源。全面推行土地、水源、海域等不可再生资源的有偿开发和保护性利用，推进集约化生产和清洁生产，大力发展循环经济，逐步构建节约型的产业结构和消费结构。加快贵屿镇废旧电子信息产品拆解处理示范工程建设，把废旧电器回收拆解引导到产业化经营上来。

加强环境保护和治理。着重推进治污保洁工程、重点工业污染源全面达标、重点流域区域环境综合整治。推动龙珠水质净化厂市场化运作，动工建设北轴污水处理厂一期工程、潮阳区污水处理厂和潮南区2个污水处理厂，加快建设澄海区清源水质净化厂、南澳县2个污水处理工程和雷打石等6个生活垃圾卫生填埋场。尽快出台汕头市生活饮用水源保护条例，加强对韩江、榕江流域水质保护，加快练江整治。完善城市管理体制，提高城市管理水平，改善市容市貌。

加快生态建设步伐。继续开展生态城市、生态示范区、生态社区、生态镇、生态村和生态工业园区创建活动，加大对湿地红树林、水源涵养林、水土保持林、沿海沙滩防护林、农田防护林、自然保护区以及南澳国家森林公园、潮南大南山省级森林公园的建设管护力度。落实“绿色通道建设规划”，构建绿色生态景观。坚持不懈抓好殡葬改革，保护生态环境。

加大计划生育管理力度。进一步落实人口与计划生育目标管理责任制，坚决兑现对各级计生责任人的奖惩。搞好计生基层基础建设，重点抓好纯二女户结扎、依法征收社会抚养费和查处党员干部违法违规生育工作。加强流动人口计生工作管理和服务，提高计划生育管理与服务水平。

十、大力发展文化事业，加快社会各项事业发展

提升公民现代文明素质和城乡文明程度。深入学习贯彻“三个代表”重要思想，深入开展“爱国、守法、诚信、知礼”现代公民教育，大力弘扬新时期汕头人精神，提高公民思想道德素质。加强未成年人思想道德建设。普及科学知识，倡导科学文明的生活方式。继续开展全民读书活动，深入开展群众性精神文明创建活动。

推进文化大市建设。贯彻实施加快文化大市建设的决定，发展繁荣文化事业，培育发展文化产业。加大文化设施建设力度，加强基层文化建设。加快林百欣图书馆装修工程进度，争取尽快启用。优化整合文化资源，支持开展潮汕历史文化研究，振兴潮剧等优秀潮汕传统文化，培育汕头文化品牌。建立与市场经济相适应的文化管理体制，加强文化市场综合执法，繁荣文化市场。

促进教育事业协调发展。进一步巩固提高“普九”成果，突出抓好农村基础教育，积极改造薄弱学校，推动中小学校舍建设和布局调整工作。大力扩大高中办学规模，继续推进等级学校建设，增加优质学校和学位。加快汕头一中新校区建设。推进教育信息化建设和新课程实施工作，加强师资队伍建设，提高教育教学质量。积极发展职业教育和成人教育，支持民办教育发展。办好汕头职业技术学院，继续支持办好汕头大学。

提高科技创新能力。支持企业建立技术研究与开发机构，培育和搭建行业性、区域性科技创新平台，建立和完善科技中介服务体系，办好科技项目孵化基地，加速科技成果的推广转化。整合资源，大力发展科技园区和软件园。加强知识产权保护。

加强人才队伍建设。实施人才兴市战略，推进人才结构调整，改善人才创业环境，建设一支与汕头经济社会发展相适应的高素质人才队伍。建立健全人才使用与保障的机制，做好人才培养、引进和使用工作，促进人才合理流动。

发展卫生、体育等各项事业。加快建设市疾控中心，完善公共卫生体系。加强对重大传染病的防治，提高应对各类突发公共卫生事件的能力。加快城镇和农村医疗卫生体制改革，引导农民参加农村新型合作医疗。发展妇女儿童事业，做好老龄工作。启动市特殊教育学校建设前期工作，依法做好分散按比例安排残疾人就业和残疾人就业保证金征缴工作，推进残疾人事业发展。加强国防动员和国防教育工作，抓好民兵和预备役建设，做好拥军优属、优抚安置工作。广泛开展全民健身活动，推进社区公益性体育设施建设。加快中国乒乓球训练基地建设步伐，积极备战第十二届省运会。重视和做好宗教、对台、人防、外事、侨务、档案、保密、方志等工作。

十一、关注民生，构筑和谐汕头

努力扩大就业再就业。落实就业再就业工作目标责任制，兑现小额担保贷款、社会保障补贴等优惠政策，促进下岗失业人员就业再就业。加强农民职业培训，推进城乡统筹就业。建立公共就业服务管理制度，加快劳动力市场信息网络建设。开拓劳务市场，做好劳务输出。加强职业技能培训，大力发展技工教育。严格执行最低工资制度，建立欠薪预警机制，加强企业工资支付监控。加大劳动合同管理和劳动监察执法力度，维护劳动者合法权益。

加快完善社会保障体系建设。巩固和扩大社会保险覆盖面，不断完善养老、失业、工伤、生育、医疗保险、最低生活保障制度，探索建立企业补充养老、被征地农民养老保障办法，建立健全多层次的社会保障体系。强化基金征缴和监督，加大财政投入，增强保障能力。加快企业退休人员社会化管理步伐，推进社会保障信息化建设。

解决好群众关注的热点难点问题。大力推进农村水改、安居、扶贫济困等“十项民心工程”，切实解决部分群众饮水难、入学难、打官司难、住房难、看病难等问题。继续开展清理拖欠工程款和农民工工资等工作。妥善解决“五保户”和特困户的生活困难，做好社会救助工作。深入开展打击假冒伪劣食品、药品、卷烟、农资等违法犯罪活动，强化食品卫生监督，实施“放心肉”工程，确保人民群众身体健康。

切实维护社会稳定。保持高压态势，严厉打击各种社会黑恶势力和凶杀绑架等严重刑事犯罪。加大力度打击“两抢两盗”，坚决打击“六合彩”等赌博活动。建立健全社会安全预警机制，加强社会治安综合治理，增强城乡居民的安全感。认真做好群众来信来访、人民调解工作，努力化解社会矛盾。落实安全生产责任制，加大安全生产执法力度，消除各种事故隐患。加强国家安全工作，依法打击法轮功邪教组织，切实维护国家安全和社会政治稳定。

切实加强政府自身建设

加强政府自身建设，是正确履行政府职责、全面完成全年工作任务的重要保证。按照科学行政、民主行政、依法行政的要求，全面加强政府行政能力建设，努力建设廉洁务实高效政府、法治政府和人民满意政府。

加强民主制度建设。坚持向人大及其常委会报告工作和向人民政协通报情况的制度。自觉接受人大及其常委会和人民政协的监督，高度重视人大代表、政协委员的建议、提案办理工作，办好人大议案。认真听取民主党派、工商联、无党派人士和各人民团体的意见。接受新闻舆论和公众监督。推进政务、厂务和村务公开，保障人民的知情权、参与权和监督权。认真组织好村居委会的换届选举工作，保证人民依法直接行使民主权利，管理基层公共事务和公益事业。

深化行政管理体制改革。转变政府职能，加快政企、政事、政资分开，着力在规范市场准入、强化市场监管、健全社会信用体系等方面为市场主体服务。进一步简政放权，进行第二批市、区管理权限调整。培育和规范行业协会、中介组织。创新行政决策机制，推进政府决策科学化、民主化、法制化，建立和完善决策责任追究制度，完善社情民意反映制度、重大事项社会公示听证制度、法律审查制度和专家咨询论证制度，扩大政府决策及实施的社会基础。

增强政府依法管理经济和社会的能力。贯彻落实《全面推进依法行政实施纲要》，改进政府管理经济的方式方法，提高政府领导经济工作的能力。依法管理和规范社会事务，建立健全社会利益协调机制，建立和完善社会矛盾化解机制，提高依法处置和化解矛盾的能力。建立健全突发事件应急机制，提高政府保障公共安全和处置突发事件的能力。加强和规范行政执法，做好行政复议工作，落实行政执法责任制和执法行为评议考核制。提高政府立法质量。发挥法律服务在经济发展和社会稳定中的作用。探索建立政府法律顾问制度。完成“四五”普法任务，营造崇尚法律的良好氛围。

推进政风建设。以提高行政效能建设为突破口，在行政机关党员中全面开展“理想、责任、能力、形象”教育，努力建设一支政治坚定、业务精通、清正廉洁、作风优良的公务员队伍。强化责任意识和大局意识，提倡敢于负责的精神，主动迎难解决问题。增强各级公务员的服务意识，更好地为发展服务、为基层服务、为群众服务。进一步改进和完善政绩考核办法，探索建立政府绩效评估体系。加快电子政务建设步伐，推进网上并联审批。积极推进惩治和预防腐败体系的建设，强化行政效能监察，落实廉政建设责任制，依法严肃查处违纪违法案件。

各位代表，今年是届中之年，处于承上启下的关键阶段，做好今年各项工作十分重要。让我们紧密团结在以胡锦涛同志为总书记的党中央周围，高举邓小平理论和“三个代表”重要思想伟大旗帜，树立和落实科学发展观，开拓进取，扎实工作，为实现汕头经济社会跨越式发展而努力奋斗！
